# CETP gene polymorphisms and risk of coronary atherosclerosis in a Chinese population

**DOI:** 10.1186/1476-511X-12-176

**Published:** 2013-11-27

**Authors:** Jun Wang, Li Jun Wang, Yong Zhong, Ping Gu, Jia Qing Shao, Shi Sen Jiang, Jian Bin Gong

**Affiliations:** 1 Departments of Cardiology, School of Medicine, Nanjing University, Jinling Hospital/Nanjing General Hospital of Nanjing Military Command, 305 Zhongshan East Road, Nanjing, Jiangsu Province 210002, China; 2 Departments of Health-Care, School of Medicine, Nanjing University, Jinling Hospital/Nanjing General Hospital of Nanjing Military Command, 305 Zhongshan East Road, Nanjing, Jiangsu Province 210002, China; 3 Departments of Endocrinology, School of Medicine, Nanjing University, Jinling Hospital/Nanjing General Hospital of Nanjing Military Command, 305 Zhongshan East Road, Nanjing, Jiangsu Province 210002, China

**Keywords:** Coronary atherosclerosis, CETP, Genetic mutation, HDL-C

## Abstract

**Background:**

Coronary atherosclerosis, the most common form of coronary artery disease (CAD), is characterized by accumulation of lipid in the walls of coronary arteries. Recent data from clinical trials have showed that high-density lipoprotein cholesterol (HDL-C) has causal role in the pathogenesis and development of coronary atherosclerosis. Cholesteryl ester transfer protein (CETP) is an important regulator of plasma HDL-C. Several genetic mutations in the CETP gene were found to be associated with HDL-C levels. The aim of the present study is to evaluate the association of HDL-C-related CETP polymorphisms and risk of coronary atherosclerosis.

**Methods:**

We investigated the association of seven single nucleotide polymorphisms (SNP) (rs1800775, rs708272, rs5882, rs1532624, rs1864163, rs7499892, and rs9989419) in the CETP gene with the risk of coronary atherosclerosis and levels of HDL-C in a case–control study in China. Included in the study were 420 patients with coronary atherosclerosis and 424 healthy controls. SNP genotyping was performed by TaqMan allelic discrimination assay and serum lipid levels were measured by standard laboratory methods.

**Results:**

Carriers of the AA and GA + AA genotypes of rs708272 had significant lower risks of coronary atherosclerosis (OR = 0.55, 95% CI: 0.36-0.85, p = 0.003; OR = 0.67, 95% CI: 0.50-0.90, p = 0.007, respectively) compared to those with GG genotype. These relations remained significant after adjustment for confounding effects of age, smoking, diabetes and hypertension. The rs1800775 polymorphism was significantly associated with serum levels of HDL-C in healthy controls (p = 0.04). Besides, rs708272 was in close linkage disequilibrium (LD) with rs1800775 in this study.

**Conclusions:**

Our findings indicated that CETP rs708272 may be associated with the risk of coronary atherosclerosis and rs1800775 may influence serum HDL-C levels in healthy controls in Chinese.

## Background

Coronary atherosclerosis, a chronic inflammatory disease characterized by the accumulation of fatty materials such as cholesterol and triglyceride on the walls of the coronary arteries, is the principal cause of coronary artery disease (CAD) [1,2]. HDL is believed to be a protective factor against CAD, and the inverse relationship between plasma HDL-C and the incidence of CAD is well established [3,4]. Preliminary studies have suggested that HDL infusions can induce atherosclerosis regression [5]. Protective effect of HDL on atherosclerosis may due to its role in preventing oxidation or other adverse effects of low-density lipoprotein cholesterol (LDL-C) on endothelial cell, moreover, HDL also can directly stimulate endothelial cell to produce nitric oxide, beneficial anti-inflammatory, anti-apoptotic and anti-thrombotic agents as well as promote endothelial repair processes [6,7].

Cholesteryl ester transfer protein (CETP) is a hydrophobic glycoprotein, which has an established role in transporting of cholesterol from the peripheral tissues to the liver for elimination through exchanging triglycerides of VLDL and LDL against cholesteryl esters of HDL. The possibility that increased function of CETP might be proatherogenic and that inhibition of its activity might be antiatherogenic was first raised >20 years ago [8]. CETP inhibitors as novel drugs have been developed to raise HDL-C concentrations and improve HDL function in patients with coronary disease, although the effect and safety still need to be confirmed [9].

Several mutations in the CETP gene have been identified as a cause of CETP deficiency and change of HDL-C levels, but the associations of these single nucleotide polymorphisms (SNP) and susceptibility to atherosclerosis still lacks consistency [10-12]. Besides, the relation between these SNPs and risk of coronary atherosclerosis has not been fully studied in Chinese population.

To help clarify whether the CETP SNPs which were previously shown to be associated with plasma HDL-C levels and also confirmed in a genome-wide association study [10,13-17] are associated with susceptibility of coronary atherosclerosis and plasma HDL-C levels, we examined seven SNPs in the CETP gene (rs1800775, rs708272, rs5882, rs1532624, rs1864163, rs7499892, and rs9989419) in a case–control study in Chinese population.

## Results

Our study population consisted of 420 cases and 424 healthy controls. Characteristics of the study subjects are shown in Table 1. Cases and controls were comparable with respect to age and gender. Cases were more probably to smoke cigarettes (50.9% vs. 32.3%), have diabetes (21.0% vs. 12.0%) and hypertension (48.7% vs. 38.7%). Besides, cases have significant lower levels of serum HDL-C and higher levels of serum total cholesterol (TC) and LDL-C than that in controls.

**Table 1:** Selected characteristics of cases and controls

**Characteristics**	**Cases (n = 420)**	**Controls (n = 424)**	**P value**
Age (year)	66(61–70)	66(60–70)	0.97
Sex (Male/female)	167/253	168/256	0.52
Smoking (Yes/no)	214/206	137/287	<0.001
Diabetes (Yes/no)	88/331	51/372	0.001
Hypertension (Yes/no)	205/215	164/260	0.003
BMI (kg/m^2^)	24.3(23.0-25.9)	24.2(22.9-26.6)	0.86
Total cholesterol (mmol/L)	4.72(4.11-5.48)	4.09(3.44-5.22)	<0.001
HDL-C (mmol/L)	1.20(1.09-1.51)	1.23(1.01-1.67)	<0.001
LDL-C (mmol/L)	2.83(2.47-3.29)	2.45(2.06-3.13)	<0.001

The associations of CETP variants with risk of coronary atherosclerosis are presented in Table 2. The genotype distributions of these seven variants showed no deviation from the expected Hardy-Weinberg equilibrium among controls (p > 0.05). Of these SNPs, carriers of the AA and GA + AA genotypes of rs708272 had significant lower risk of coronary atherosclerosis (OR = 0.55, 95% CI: 0.36-0.85, p = 0.003; OR =0.67, 95% CI: 0.50-0.90, p = 0.007, respectively) compared with carriers of the major genotype. These associations remained statistically significant after further adjustment for age, smoking, hypertension and diabetes. None of the other SNPs examined was associated with coronary atherosclerosis.

**Table 2:** Association of genetic variants in CETP gene with risk of coronary atherosclerosis

**SNP**	**Controls, n**	**Cases, n**	**OR (95% CI)**	**P for trend**
rs1800775				
AA	116	102	1.00	
AC	222	216	1.11(0.79-1.56)	
CC	83	100	1.43(0.93-2.19)	0.32
AC + CC	305	316	1.19(0.86-1.65)	
rs1532624				
CC	199	209	1.00	
CA	191	183	0.88(0.66-1.18)	
AA	34	29	0.80(0.45-1.41)	0.52
CA + AA	225	212	0.87(0.65-1.16)	
rs708272				
GG	139	176	1.00	
GA	207	192	0.71(0.52-0.97)	
AA	74	50	0.55(0.36-0.85)	0.04
GA + AA	281	242	0.67(0.50-0.90)	
rs1864163				
GG	289	282	1.00	
GA	116	115	1.05(0.76-1.44)	
AA	18	23	1.49(0.75-2.93)	0.92
GA + AA	134	138	1.10(0.81-1.49)	
rs7499892				
GG	261	259	1.00	
GA	136	138	1.06(0.78-1.45)	
AA	27	24	0.96(0.53-1.75)	0.88
GA + AA	163	162	1.05(0.78-1.40)	
rs5882				
AA	142	135	1.00	
AG	219	215	1.00(0.73-1.37)	
GG	63	71	1.13(0.73-1.74)	0.89
AG + GG	282	286	1.03(0.76-1.39)	
rs9989419				
GG	198	201	1.00	
GA	185	179	0.96(0.71-1.29)	
AA	41	41	0.96(0.59-1.58)	0.71
GA + AA	226	220	0.96(0.72-1.27)	

ORs: adjusted for age.

Four SNPs in the CETP gene (rs1800775-rs1532624-rs708272-rs1864163) were in linkage disequilibrium with D’ ranging from 0.66 to 1.00 and r^2^ ranging from 0.07 to 0.55. However, we did not find any CETP haplotype that was significantly associated with risk of coronary atherosclerosis (data not shown).

Finally, we investigated the associations between the seven SNPs and serum HDL-C levels in 424 healthy controls. In univariate analyses, CETP rs1800775 was significantly associated with decreased serum level of HDL-C (p = 0.04). Carriers of the mutant alleles of rs1800775 (A/C: 1.45 ± 0.62 mmol/L; C/C: 1.28 ± 0.41 mmol/L) had significantly decreased serum HDL-C levels compared with subjects with GG genotype (1.49 ± 0.72 mmol/L). When ANCOVA model was applied, rs1800775 still showed significant association with HDL-C level (p = 0.04). None of the other studied SNPs was associated with serum HDL-C level (Table 3).

**Table 3:** Association between CETP polymorphisms and serum HDL-C levels in health control subjects

**SNP**	**M/m**	**HDL-C (mmol/L) mean ± SD**			**P**
		**MM**	**Mm**	**mm**	
rs1800775	A/C	1.49 ± 0.72	1.45 ± 0.62	1.28 ± 0.41	0.04
rs1532624	C/A	1.38 ± 0.51	1.45 ± 0.68	1.54 ± 0.80	0.27
rs708272	G/A	1.34 ± 0.55	1.46 ± 0.62	1.46 ± 0.70	0.15
rs1864163	G/A	1.43 ± 0.65	1.44 ± 0.57	1.22 ± 0.39	0.39
rs7499892	G/A	1.46 ± 0.65	1.39 ± 0.54	1.27 ± 0.64	0.23
rs5882	A/G	1.39 ± 0.61	1.46 ± 0.66	1.38 ± 0.48	0.49
rs9989419	G/A	1.46 ± 0.67	1.39 ± 0.57	1.42 ± 0.59	0.55

M indicates major allele; m indicates minor allele.

## Discussion

Considering the crucial role of CETP in lipid metabolism, we investigated the association of seven SNPs in this gene and the risk of coronary atherosclerosis in a Chinese population. Our results showed that rs708272 polymorphism may reduce the risk of coronary atherosclerosis and rs1800775 mutation may decrease serum HDL-C levels in healthy controls in our population.

The human CETP gene is located on chromosome 16q21, and several mutations in this gene have been reported to alter the function of CETP and plasma HDL-C levels [10,13]. Among these SNPs, CETP TaqIB site (rs708272) is the most studied one. The results of prospective Women’s Genome Health Study (WGHS), conducted in American women, showed that carriers of B2B2 and B1B2 genotypes (56 mg/dL and 52 mg/dL) had significant higher levels of plasma HDL-C than carriers of B1B1 genotype (50 mg/dL) [10]. Moreover, a meta-analysis, based on the data from year 1970 to 2008, also found that individuals with TaqIB B2 allele had higher mean HDL-C concentrations and lower mean CETP activity compared with carriers of B1 allele [13]. Besides, the relationship between TaqIB polymorphism and HDL-C levels was also confirmed by other studies [18,19]. But the association between CETP TaqIB and CAD risk lacks consistency. A case–control study among Singapore population [20] found that the absence of B2 allele was associated with 2.0-fold increased risk of CAD, but a meta-analysis revealed that B2B2 genotype had a 1.45-fold significant increased risk of CAD compared with B1B1 genotype in population-based studies [21]. In our study, we also found that B2B2 genotype conferred a significant reduced risk of coronary atherosclerosis which had the same directions of risk with the previous study done in Singapore population. In addition, controls with B1B2 or B2B2 genotype had non-significant higher level of serum HDL-C (1.46 ± 0.62 mmol/L and 1.46 ± 0.70 mmol/L) compared with the controls with B1B1 genotype (1.34 ± 0.55 mmol/L), which was in accordance with the WGHS results. CETP TaqIB is located in intron 1 of CETP gene, and the functional phenotype of this mutation is still unclear.

CETP -629C/A (rs1800775) is located in promoter region of the CETP gene, and this variation may influence the functionality of CETP gene by changing in binding site Sp1/Sp3 and in turn repress CETP promoter activity [22,23]. Rs1800775 was found to be significantly associated with plasma HDL-C levels in the WGHS with average HDL-C (mg/dL) of 49 in CC carriers, 52 in CA carriers and 55 in AA carriers [10]. The same tend of association was also found among Latvian population [24]. In our study, healthy controls carrying the CC genotype (1.28 ± 0.41 mmol/L) had significant lower level of HDL-C compared with subjects with AA genotype (1.49 ± 0.72 mmol/L), which was consistent with previous reports. Besides, we found CC genotype conferred a non-significant elevated risk of coronary atherosclerosis (OR = 1.43, 95% CI = 0.93-2.19). Rs1800775 was in high linkage disequilibrium with rs708272 in our study (D’ = 0.97, r^2^ = 0.55), and this association was also suggested by several other studies [10,25].

The exact mechanisms which may link CETP genetic polymorphism to coronary atherosclerosis are largely unclear. But the linkage between CETP polymorphisms and plasma HDL-C level may provide a possible mechanism that merits further investigation. In addition, the limitation of this study was the relatively small sample size, which hampered our ability to detect some significant associations.

## Conclusions

In conclusion, the present study demonstrating that the A allele of the rs708272 may confer decreased risk of coronary atherosclerosis, and rs1800775 may exert effect on serum HDL-C levels in Chinese population. Future studies with larger sample sizes as well as functional studies are needed to confirm our findings.

## Methods

### Study subjects

A case–control study including 420 consecutive patients with coronary atherosclerosis and 424 healthy controls was carried out. Cases were collected from Jinling hospital in Nanjing between June 2008 and May 2012. Clinical diagnosis of coronary atherosclerosis was evaluated by percutaneous coronary angiography, reviewed by two experienced cardiologists. Healthy control subjects, without coronary atherosclerosis, were selected in the same period and from the same hospital, and were frequency matched to the cases by age (5-year age groups) and gender. All controls were individuals free of CAD that determined by medical history, clinical examinations, or electrocardiography. Those with coronary myocardial bridge were excluded from the study. At enrollment, demographic characteristics, anthropometric measures, medical histories were collected from each subject by a trained interviewer using a structured questionnaire. Written informed consent was obtained from all enrolled participants and this study was approved by the Ethics Committee of Jinling hospital.

### Laboratory tests

Blood samples were drawn for measurement of serum levels of TC, HDL-C, LDL-C after a 12-hour overnight fast. Serum levels of TC (mmol/L), HDL-C (mmol/L), and LDL-C (mmol/L) were determined by colorimetric enzymatic assays with use of an Auto-Analyzer (AU 2700 Olympus, FirstChemical Ltd, Tokyo, Japan).

### Genotyping

Genomic DNA was extracted from peripheral blood leucocytes using Promega DNA Extraction Kit (Promega, Madison, WI, USA). Genotyping was performed using the TaqMan assay on the ABI PRISM 7900HT Sequence Detection System (Applied Biosystems, Foster City, CA, USA), in a 384-well format, with dual fluorescent reporter probes VIC and FAM. The genotyping call rate was >95%, and the completion rate was >99%. The quality and potential misclassification of the genotyping were assessed by re-evaluating 5% of duplicate DNA samples (42 total samples) that were randomly selected from the whole population and placed within the same reaction plates used for the study subjects. The concordance rate for the quality control samples was 100%.

### Statistical analysis

We used SAS software (version 9.3; SAS Institute, Inc.) for the statistical analyses. χ^2^ statistics and the t test were used to evaluate case–control differences in the distribution of risk factors. Variables were tested for normality with Shapiro-Wilk statistics. Skewed data, including age, BMI, TC, HDL-C and LDL-C were log transformed and expressed as medians and interquartile ranges. The odds ratios (ORs) and 95% confidence intervals (CIs) for the associations between the SNPs and disease risk were estimated by unconditional logistic regression. Hardy-Weinberg equilibrium for genotypic distribution and linkage disequilibrium between loci were assessed by HaploView version 4.0 (Daly Lab at the Broad Institute, Cambridge, MA, USA) [26]. Associations between haplotypes (> 1% frequency) and the risk of coronary atherosclerosis were evaluated by computing OR and 95% CI using HAPSTAT, assuming an additive model, using the most common haplotype as the referent category [27]. Both univariate ANOVA and multivariate ANCOVA analyses adjusting for age, smoking, diabetes and hypertension were performed to determine the effects of the CETP polymorphisms on serum HDL-C levels. A two tailed P-value of 0.05 was considered statistically significant.

## Abbreviations

CAD: Coronary artery disease; HDL-C: High-density lipoprotein cholesterol; LDL-C: Low-density lipoprotein cholesterol; CETP: Cholesteryl ester transfer protein; SNP: Single nucleotide polymorphisms; TC: Total cholesterol; CHB: Han Chinese in Beijing; BMI: Body mass index.

## Competing interests

The authors declare that they have no competing interests.

## Authors’ contributions

JW and LJW designed the molecular genetic studies and drafted the manuscript. YZ and PG carried out the genotyping experiments. JQS performed the statistical analysis. SSJ and JBG participated in the design of sample collection and improved the manuscript. All authors read and approved the final manuscript.
